# Supplementary material for: Development of Oral Microemulsion Spray Containing Pentacyclic Triterpenes-Rich Centella asiatica (L.) Urb. Extract for Healing Mouth Ulcers
Source: Pharmaceutics. 2022 Nov 20;14(11):2531. doi: 10.3390/pharmaceutics14112531 (PMC9694358; doi:10.3390/pharmaceutics14112531)
Supplement: Supplementary file 1 [file pharmaceutics-14-02531-s001.zip › pharmaceutics-1991678-SM.pdf]

Supplement data

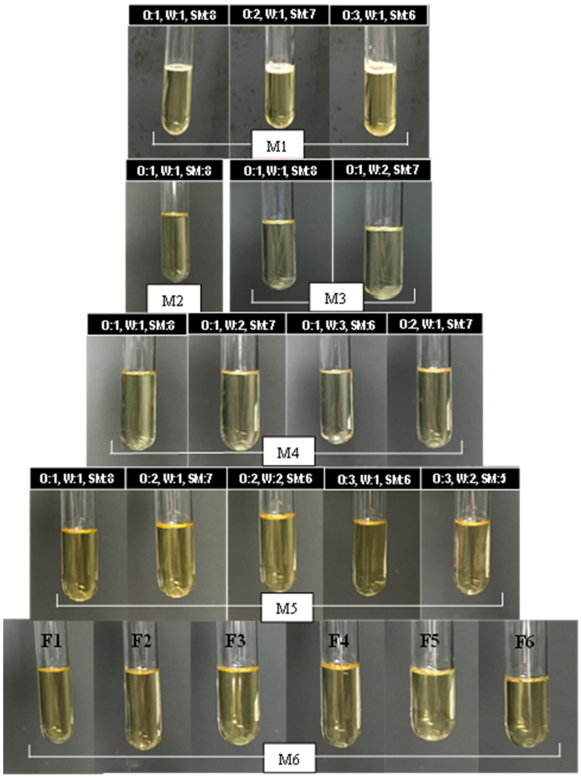

Supplement Data S1. The formulations of microemulsion from the pseudo-ternary phase diagram M1-M6 (W = water, O = oil, and SM = surfactant mixture)

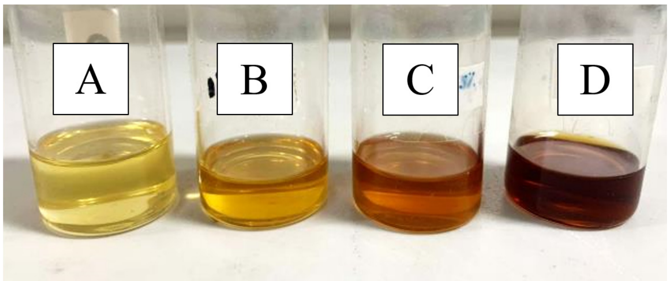

Supplement Data S2. The formulations of microemulsion base and microemulsion containing 1, 2.5 and 5% PRE (A = microemulsion base, B = microemulsion containing 1% PRE, C = microemulsion containing 2.5% PRE and D = microemulsion containing 5% PRE)

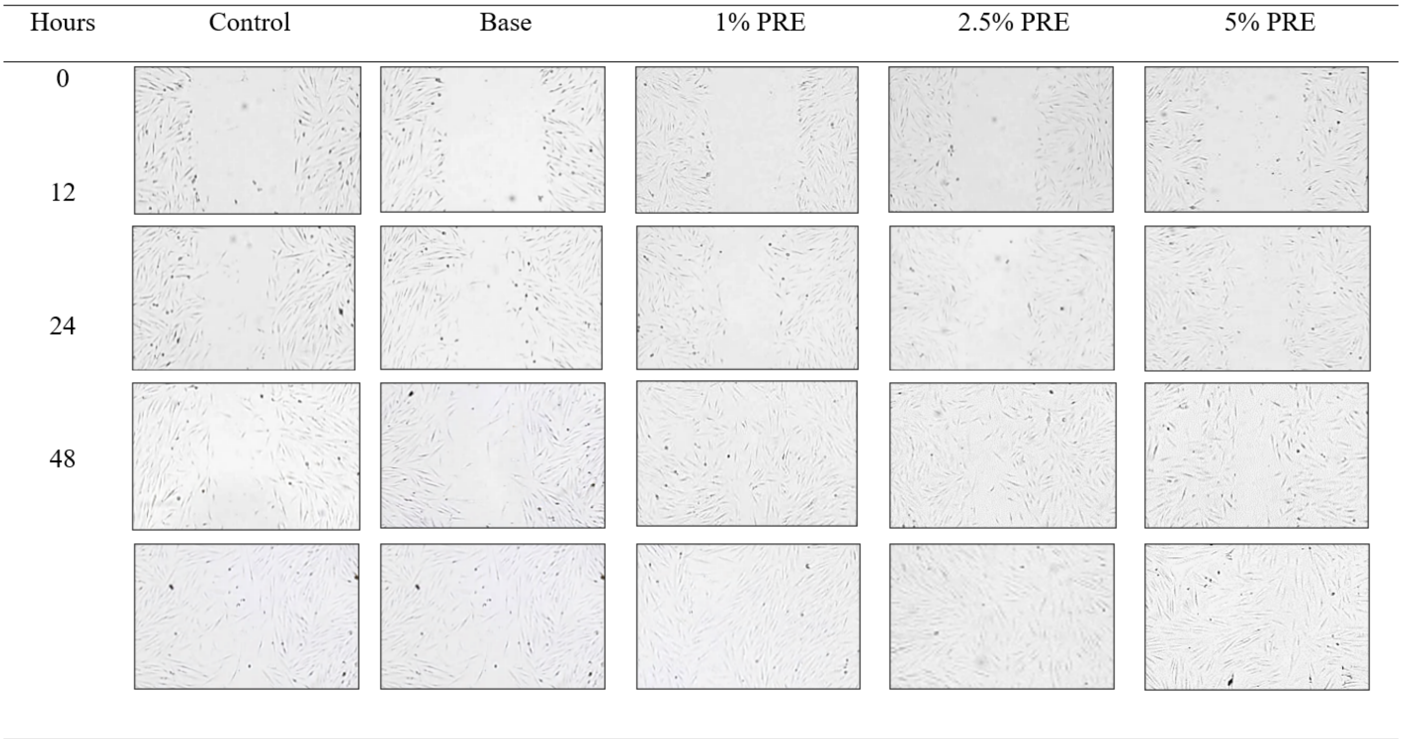

Supplement Data S3. Effect of microemulsion base and microemulsion containing PRE at various concentrations on HDF cells migration assay (base = microemulsion base, 1%PRE, 2.5%PRE, and 5%PRE = microemulsion containing 1, 2.5, and 5% PRE, respectively

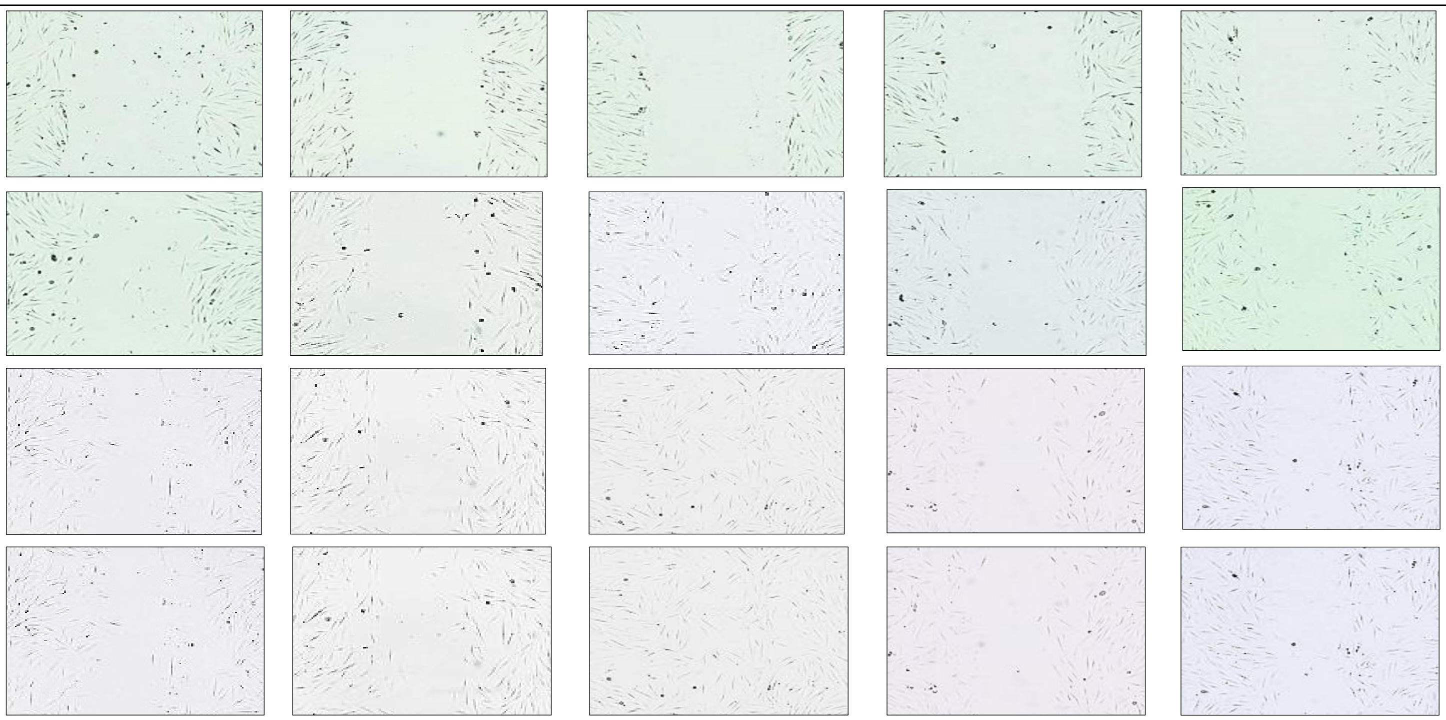

Supplement Data S4. Effect of microemulsion base and microemulsion containing PRE at various concentrations on HGF cells migration assay (base = microemulsion base, 1%, 2.5%, and 5%PRE = microemulsion containing 1, 2.5, and 5% PRE, respectively.
